# The high molecular weight neurofilament subunit plays an essential role in axonal outgrowth and stabilization

**DOI:** 10.1242/bio.20149779

**Published:** 2014-09-26

**Authors:** Sangmook Lee, Thomas B. Shea

**Affiliations:** Center for Cellular Neurobiology and Neurodegeneration Research, Department of Biological Sciences, University of Massachusetts at Lowell, Lowell, MA 01854, USA

**Keywords:** Neurofilament, Axonal outgrowth, Axonal stability, Nervous system development, Cytoskeleton

## Abstract

Neurofilaments (NFs) are thought to provide structural support to mature axons via crosslinking of cytoskeletal elements mediated by the C-terminal region of the high molecular weight NF subunit (NF-H). Herein, we inhibited NF-H expression in differentiating mouse NB2a/d1 cells with shRNA directed against murine NF-H without affecting other NF subunits, microtubules or actin. shRNA-mediated NF-H knockdown not only in compromised of late-stage axonal neurite stabilization but also compromised early stages of axonal neurite elongation. Expression of exogenous rat NF-H was able to compensate for knockdown of endogenous NF-H and restored the development and stabilization of axonal neurites. This rescue was prevented by simultaneous treatment with shRNA that inhibited both rat and murine NF-H, or by expression of exogenous rat NF-H lacking the C-terminal sidearm during knockdown of endogenous NF-H. Demonstration of a role for NF-H in the early stages of axonal elaboration suggests that axonal stabilization is not delayed until synaptogenesis, but rather that the developing axon undergoes sequential NF-H-mediated stabilization along its length in a proximal–distal manner, which supports continued pathfinding in distal, unstabilized regions.

## INTRODUCTION

Establishment of appropriate connections among neurons in the developing nervous system is accomplished by detection and response to a complex series of guidance cues and ultimate target recognition, collectively referred to as “axonal pathfinding” (for a review, see Raper and Mason, 2010). During this process, a neuron must modify its axonal cytoskeleton from a relatively plastic scaffold that accommodates rapid elongation into a stabilized structure that essentially locks into place in order to maintain synaptic connections for the lifetime of the individual. This transition is mediated by complex and continued interactions among cytoskeletal elements.

Initial elaboration of putative axonal neurites requires localized depolymerization of subcortical actin filaments and polymerization of microtubules (MTs), which provide permissive and driving forces, respectively (Shea, 1990). Elaboration of exploratory neurites also requires the primordial intermediate filament, vimentin (Shea et al., 1993; Boyne et al., 1996). Continued elongation of putative axonal neurites is no longer dependent upon vimentin which is downregulated within 24 hr following axonal initiation (Shea et al., 1993; Yabe et al., 2003), but instead requires neurofilaments (NFs), which are the major intermediate filament of mature neurons (Shea and Beermann, 1999).

Intermediate filaments (IFs) in general provide mechanical strength to cells and mediate the formation of tissues (Fuchs, 1994; Bertaud et al., 2010). Perhaps nowhere can the need for long-term, stable structural support be more critical than in axons, which, once synaptogenesis has occurred, remain in place for the lifetime of the individual. NFs consist of 3 subunits, termed NF-H, NF-M and NF-L (corresponding to heavy, medium and light according to their molecular mass (Nixon and Shea, 1992; Pant and Veeranna, 1995), along with α-internexin and peripherin (Yuan et al., 2006; Yuan et al., 2012). Developmentally-delayed phospho-mediated interactions mediated by the C-terminal region of NF-H and NF-M, regulated by a complex cascade of kinase and phosphatase activity (Lee et al., 2014), are thought to link NFs with each other and with other cytoskeletal elements, and in doing so provide stability to the axonal cytoskeleton (Lewis and Nixon, 1988; Sánchez et al., 2000; Barry et al., 2007; Veeranna et al., 2008; Shea and Lee, 2011; Holmgren et al., 2012; Rao et al., 2012; Yuan et al., 2012; Shea and Lee, 2013).

Herein, shRNA-mediated depletion of NF-H compromised not only the development of stabilization following axonal outgrowth as demonstrated by resistance to retraction following colchicine treatment, but also provides inhibited axonal elaboration, indicating that NF-H facilitates relatively early stages of axonal outgrowth.

## RESULTS

### shRNA-mediated knockdown of NF-H

**Fig. 1.**
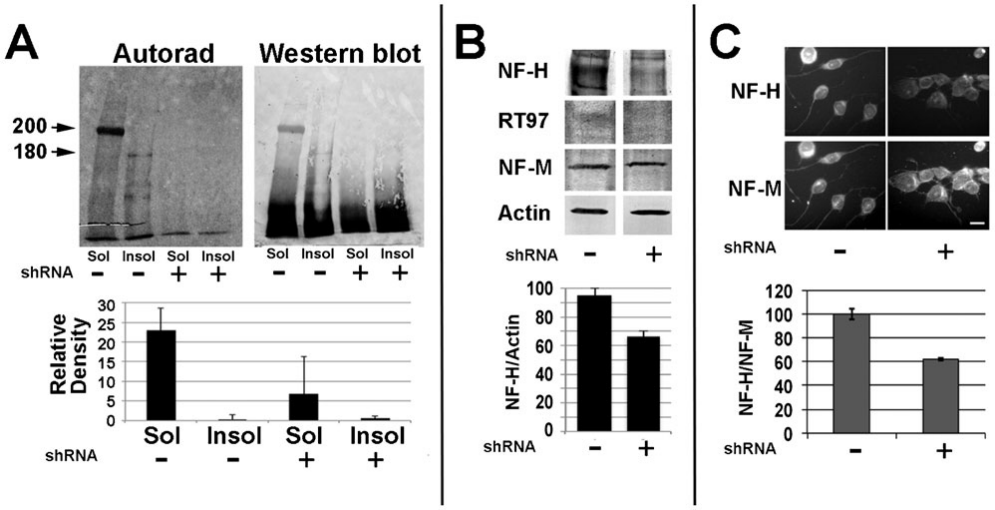
Reduction of NF-H synthesis and steady-state levels by shRNA treatment. (A) Autoradiographic and corresponding immunoblot analyses of material immunoprecipitated from Triton-soluble (“Sol”) and Triton-insoluble (“Insol”) fractions of undifferentiated cells incubated for 2 hr with ^35^S-methionine 48 hr after transfection with a cocktail of CDS1-4. The accompanying graph presents densitometric analyses of the mean (± standard error) of 200 kDa+180 kDa material in duplicate autoradiographs. NF-H synthesis and steady-state levels were significantly reduced by treatment with CDS1-4 (*p*<0.001 and *p*<0.01 for synthesis and steady-state levels, respectively; ANOVA). Additional cells transfected with CDS1-4 48 hr prior to dbcAMP-mediated differentiation were subjected to immunoblot analysis with multiple antibodies (B) or double-label immunofluorescent analyses with anti-H and anti-M (C) as indicated. The accompanying graphs present the ratio of total NF-H/actin (B) and relative intensity ratio of NF-H/NF-M as indicated (C); steady-state levels of NF-H were significantly reduced by shRNA treatment as evidenced by both immunoblot and immunofluorescent analyses (*p*<0.01 for both; Student's *t* test; panels B and C, respectively). Bar: 25 µm.

Transfection of NB2a/d1 cells with a cocktail of shRNA (CDS1-4; Table 1) reduced synthesis and steady-state levels of NF-H, without affecting levels of the other NF subunits or actin (Fig. 1). Autoradiographic analyses of NF-H immunoprecipitated from cytoskeletal and soluble fractions from cells incubated with ^35^S-methionine revealed a 68.6±11.5% reduction in radiolabel associated with NF-H (Fig. 1A). Treatment of differentiating cells with CDS1-4 reduced steady-state levels of NF-H by approximately 40% as indicated by immunofluorescent and immunoblot analyses (Fig. 1B,C).

**Table 1.**
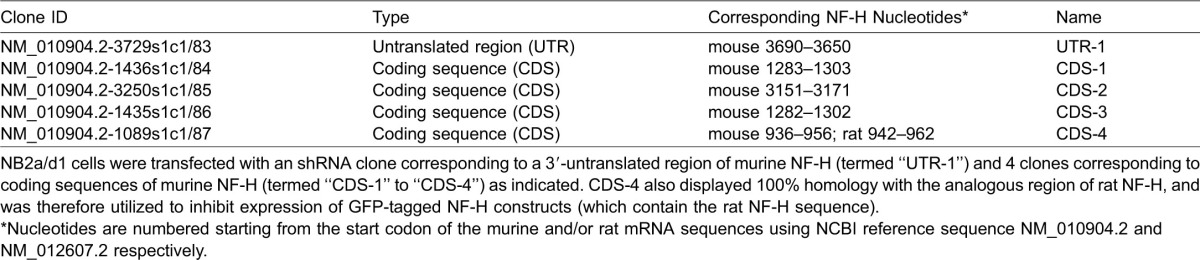
shRNA clones used in these analyses

### Knockdown of NF-H compromised axonal outgrowth and stabilization

**Fig. 2.**
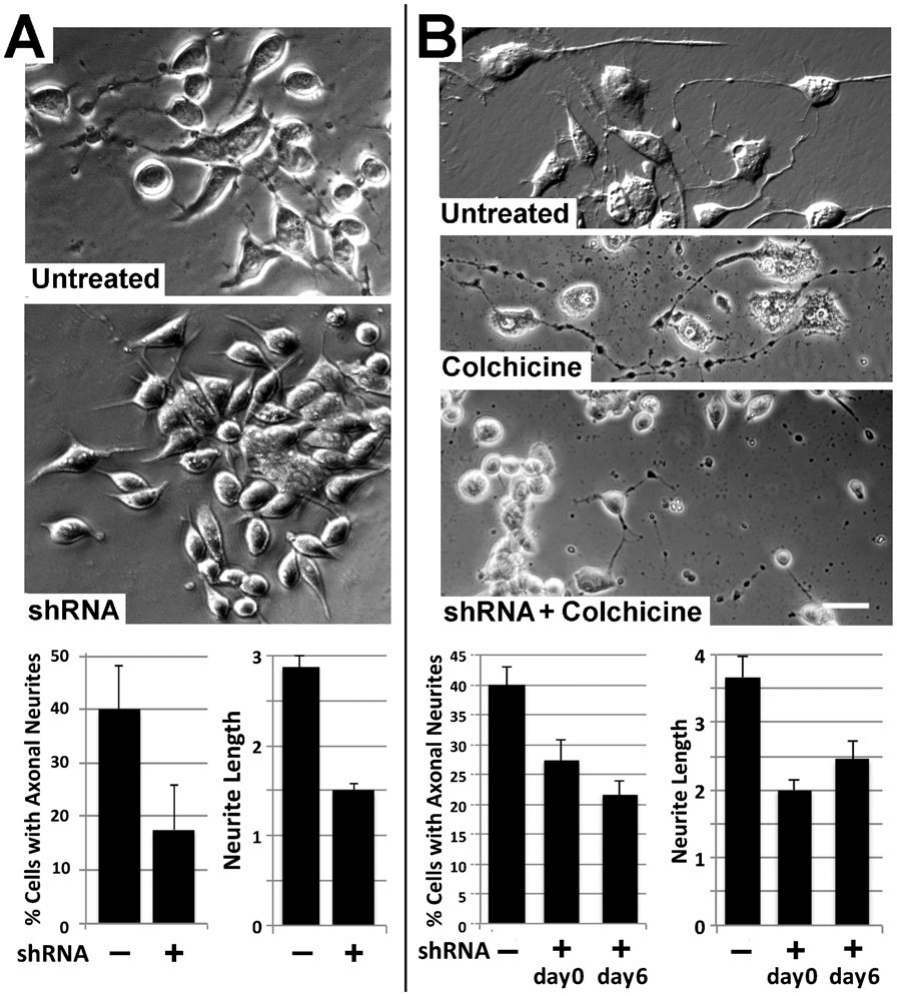
shRNA-mediated NF-H knockdown compromises axonal outgrowth and stabilization. (A) Phase-contrast images of representative fields of cells differentiated for 3 days without and with transfection with a cocktail of CDS1-4. The accompanying graphs present the mean (± standard error) the percentage of cells with axonal neurites and the length of these neurites as a function of respective somal diameters. shRNA treatment significantly reduced the percentage of cells with neurites and the length of existing neurites (*p*<0.01 for both versus untreated cultures; Student's *t* test). (B) Representative phase-contrast images of cells treated with CDS1-4 for 48 hr then differentiated for 7 days, after which alternate cultures received 1 µM colchicine for 2 hr. Colchicine treatment at this time reduces axonal neurite caliber but does not induce neurite retraction. By contrast, transfection with CDS1-4 during differentiation compromised the development of colchicine resistance. Bar: 25 µm. The accompanying graphs present the percentage of cells with axonal neurites, and the length of these neurites in respective somal diameters, following colchicine treatment without or with CDS1-4 transfection prior to differentiation (day 0) or for the final 24 hr of differentiation (day 6). Note that CDS1-4 reduced the percentage of cells with axonal neurites that resisted colchicine treatment, as well as the length of surviving neurites, when administered prior to (day 0) or following (day 6) differentiation (*p*<0.01 for both versus untreated cultures; Student's *t* test).

Since treatment with this shRNA cocktail specifically reduced NF-H levels, we next examined the impact of transfection with shRNA CDS1-4 on axonal neurite outgrowth and stabilization. Consistent with prior studies (Shea and Beermann, 1994), axonal neurites attained a mean length of 2.8±2 somal diameters after 3 days of dbcAMP treatment. However, axonal neurites attained a mean length of only 1.5±1 somal diameters in cells transfected with CDS1-4 (Fig. 2A). Axonal neurites of NB2a/d1 cells cease elongating and undergo a degree of stabilization as evidenced by development of resistance to retraction following colchicine treatment after 7 days of dbcAMP-induced differentiation (Shea and Beermann, 1994). Treatment with CDS1-4 prior to differentiation, or for the final day of this 7-day dbcAMP regimen, compromised the development of colchicine resistance (Fig. 2B). Axonal neurite length varies among cells (Shea and Beermann, 1994); quantification of the distribution of neurite lengths prior to and following shRNA treatment either during outgrowth (day 3 of dbcAMP treatment) or prior to and following colchicine treatment at day 7 demonstrated that shRNA treatment did not simply eliminate shorter neurites, but instead reduced the length of neurites of all lengths (Table 2).

**Table 2.**
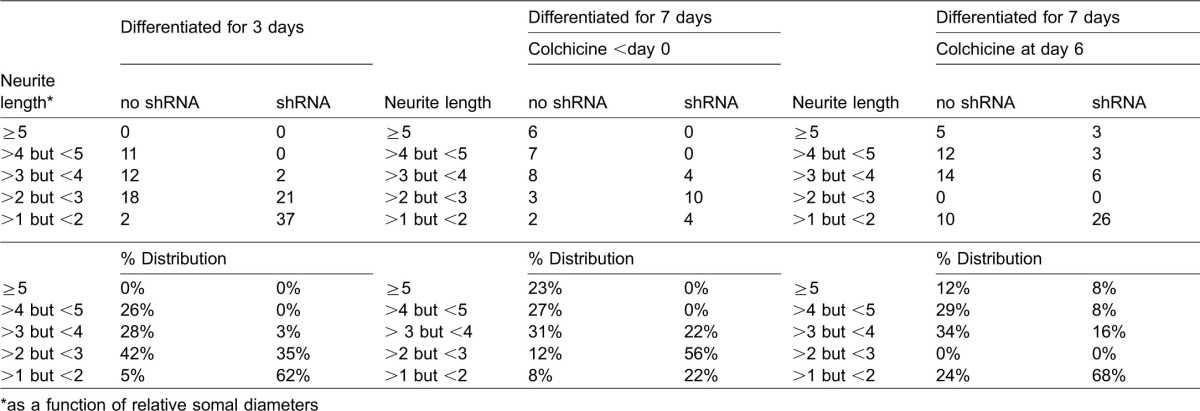
Distribution of neurite lengths in the presence and absence of shRNA directed against NF-H

### Expression of exogenous NF-H rescued axonal outgrowth

**Fig. 3.**
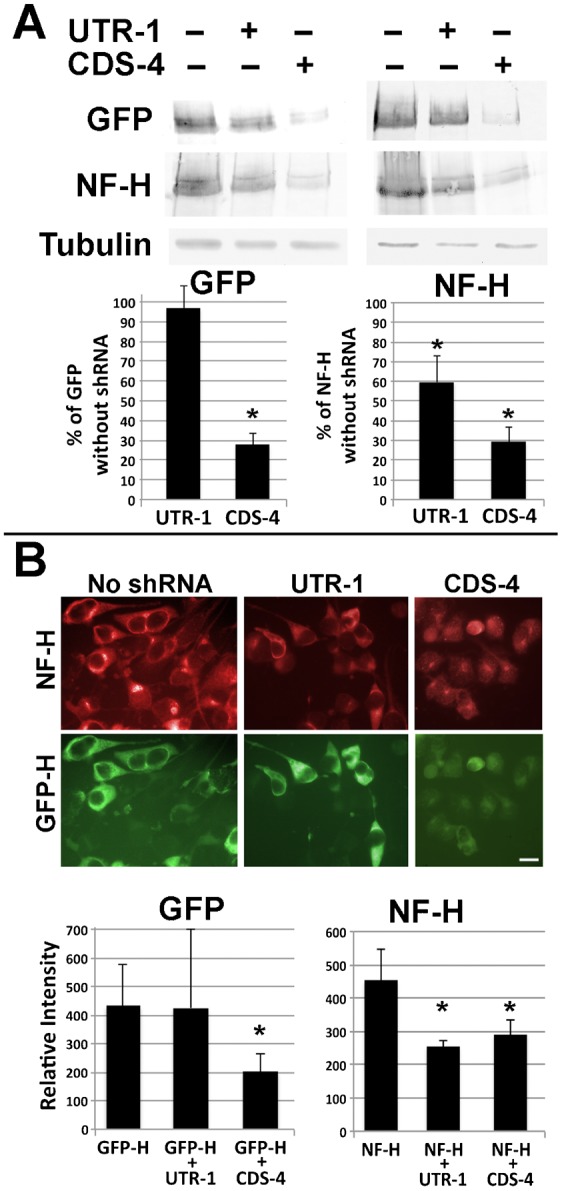
Co-transfection with GFP-H and shRNA. (A) Replicate sets nitrocellulose replicas of homogenates of cells transfected with GFP-H with and without co-transfection with UTR-1 or CDS-4 and probed with anti-GFP, anti-NF-H antibody and anti-tubulin antibody DM1A (as a loading control) as indicated. The accompanying graphs present densitometric analyses of these replicas. Co-transfection with UTR-1 did not reduce GFP-H levels but reduced endogenous NF-H as evidenced by a reduction in total NF-H immunoreactivity. Co-transfection with CDS-4 reduced both GFP-H and total NF-H immunoreactivity (asterisks indicate *p*<0.01 versus cultures not receiving shRNA; ANOVA with Fischer's post-hoc analyses). (B) Representative fluorescent images of cells transfected as in panel A and reacted with anti-NF-H followed by Texas-Red conjugated secondary antibody. Bar: 25 µm. The accompanying graphs present densitometric analyses of 20–100 cells under each condition from multiple fields. As in immunoblot analyses, co-transfection with UTR-1 did not reduce GFP-H levels but reduced total NF-H immunoreactivity. Co-transfection with CDS-4 reduced both GFP-H and total NF-H immunoreactivity (asterisks indicate *p*<0.005 versus cultures not receiving shRNA; ANOVA with Fischer's post-hoc analyses).

To provide further evidence that the above alterations in axonal dynamics were indeed due to reduction in NF-H levels, we undertook to introduce exogenous NF-H simultaneously with shRNA-mediated knockdown of endogenous NF-H. To accomplish this, cells transfected with NF-H-specific shRNA were co-transfected with GFP-tagged rat NF-H (GFP-H). Our prior studies confirm that GFP-H assembles into NB2a/d1 NFs, undergoes axonal transport and incorporates into the Triton-insoluble cytoskeleton (Lee et al., 2011; Lee et al., 2014). Transfection with GFP-H significantly raised overall levels of NF-H as ascertained by total NF-H immunoreactivity (Fig. 3A,B). To inhibit endogenous NF-H but not inhibit GFP-H expression, we co-transfected cells with a shRNA that corresponds to a 3′ untranslated region of endogenous NF-H (UTR-1) that is not contained in the GFP-tagged rat construct (Table 1). Co-transfection with UTR-1 reduced endogenous NF-H but did not reduce levels of GFP-H. (Fig. 3A,B). As a further control, additional cells expressing GFP-H were co-transfected with an shRNA (CDS-4) which shares 100% homology with both rat and mouse NF-H (Table 1), with the intent of knockdown of expression of both endogenous and exogenous (GFP-tagged) NF-H. Co-transfection with CDS-4 reduced both endogenous NF-H and GFP-H (Fig. 3A,B).

**Fig. 4.**
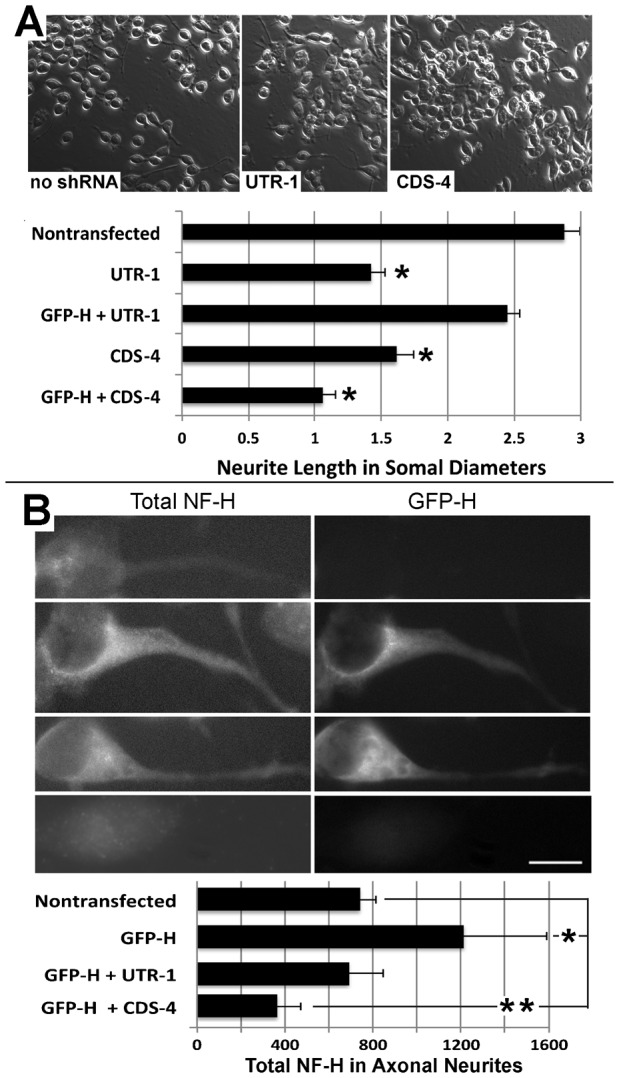
Exogenous NF-H prevents the shRNA-mediated reduction in axonal length. (A) Representative phase-contrast micrographs of cells were transfected with GFP-H without and with co-transfection with a shRNA corresponding to the untranslated region of mouse NF-H (UTR-1) or a coding sequence shared by both rat and mouse NF-H (CDS-4; Table 1). The accompanying graph presents quantification of neurite length as a function of respective somal diameters for nontransfected cells and cells transfected with GFP-H without and with co-transfection with UTR-1 or CDS-4 as indicated. Transfection with UTR-1 or CDS each reduced neurite length; cotransfection with GFP-H curtailed the reduction in neurite length mediated by UTR-1 but not by CDS-4 (asterisk indicate *p*<0.001, ANOVA with Fischer's post-hoc analyses). (B) Representative immunofluorescent images of total NF-H cells transfected as in panel A. The accompanying graph presents quantification of total NF-H within axonal neurites. A single asterisk indicates an increase (*p*<0.04) and the double asterisk indicates a decrease (*p*<0.04) in total axonal NF-H versus levels in axons of nontransfected cells; Student's *t* test). Bar: 25 µm.

We next examined whether or not expression of exogenous GFP-H could compensate for shRNA-mediated inhibition of axonal outgrowth. The mean length of neurites was reduced by 50±7% following treatment of cells with UTR-1; however, co-transfection with GFP-H curtailed the UTR-1-mediated reduction in neurite length to only 15±4% (Fig. 4A). The mean length of neurites was also reduced by and 44±8% following transfection with CDS-4; co-transfection with GFP-H could not compensate for the impact of CDS-4 on neurite length (Fig. 4A). Examination of total NF-H levels within axonal neurites confirmed that GFP-H restored levels of axonal NF-H following co-transfection with UTR-1 but not with CDS-4 (Fig. 4B).

**Fig. 5.**
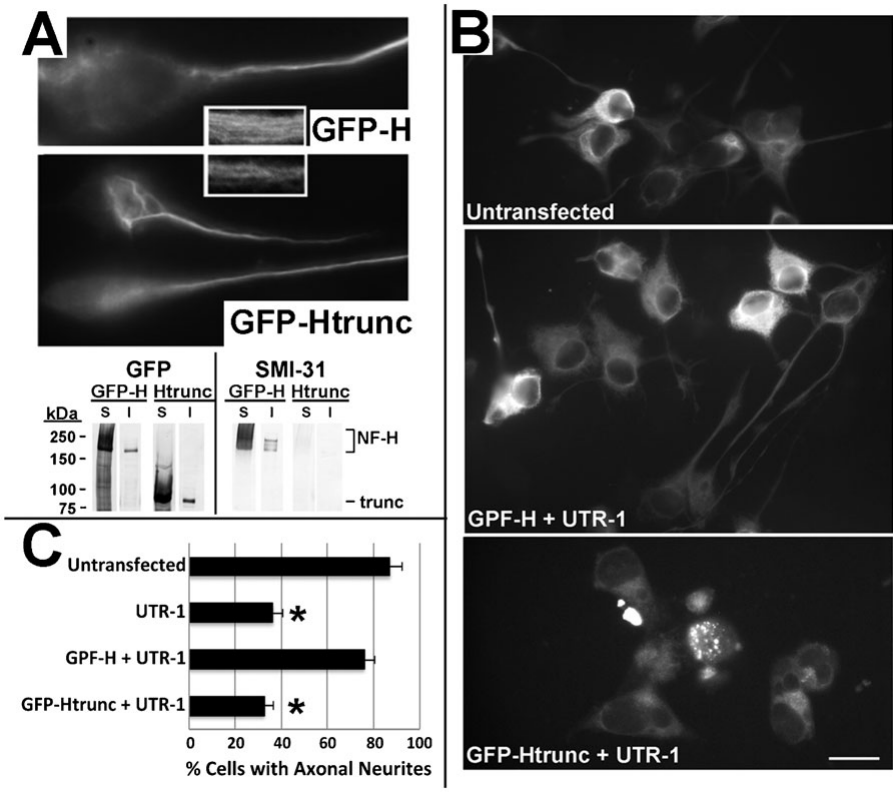
The C-terminal sidearm mediates rescue of axonal neurites by exogenous NF-H. (A) Representative epifluorescent images of differentiated cells and immunoblot analysis of Triton-soluble (S) and Triton-insoluble (I) fractions derived from cells transfected 48 hr previously with GFP-H or GFP-Htrunc as indicated. Inserts present a higher-mag of axonal NFs. Note that GFP-Htrunc is observed within filamentous profiles within axonal neurites and is recovered within the Triton-insoluble cytoskeleton. Nitrocellulose replicas were probed with anti-GFP and the NF C-terminal-specific antibody SMI-31. As anticipated, GFP-Htrunc lacks immunoreactivity with SMI-31 due to absence of the C-terminal sidearm. (B) Representative immunoflourescent images of NF-H in differentiated cells without transfection or co-transfected with UTR-1 and either GFP-H or GFP-Htrunc as indicated. (C) Quantification of the percentage of cells with axonal neurites without transfection or following transfection with UTR-1 without or with co-transfection with GFP-H or GFP-Htrunc. UTR-1 significantly reduced the number of cells with axonal neurites; as above (Fig. 4), co-transfection with GFP-H prevented this reduction, while GFP-Htrunc was unable to prevent this reduction (*p*<0.01 for both UTR-1 vs untransfected cells and UTR-1 + GFP-Htrunc vs untransfected cells; ANOVA with Fischer's post-hoc analyses). Bar: 25 µm.

NF-H is thought to provide stabilization to the axonal cytoskeleton by interacting with other NFs and other cytoskeletal elements via its C-terminal sidearm (Pant and Veeranna, 1995). We therefore reasoned that exogenous NF-H lacking the C-terminal sidearm should not be able to compensate for depletion of endogenous NF-H. As a further control that shRNA-mediated reduction in neurite length was specifically due to depletion of NF-H, we cotransfected cells with UTR-1 and GFP-tagged rat NF-H lacking the C-terminal sidearm (GFP-Htrunc). GFP-Htrunc underwent axonal transport, incorporated into filamentous structures and was recovered within the Triton-insoluble cytoskeleton (Fig. 5A). However, unlike full-length GFP-H (Fig. 4A), GFP-Htrunc was unable to rescue axonal outgrowth (Fig. 5B,C). These latter findings implicate the NF-H C-terminal sidearm in supporting axonal neurite outgrowth.

## DISCUSSION

Phospho-mediated interactions mediated by NF-H are thought to provide stability to the axonal cytoskeleton. Much of the evidence obtained *in situ*, though compelling, remains correlative and is based on the timing and distribution of phospho-NF-H; phospho-NF-H isoforms are developmentally delayed and are associated with those NFs that undergo the slowest transport and turnover within axons (Lewis and Nixon, 1988; Sánchez et al., 2000; Barry et al., 2007; Veeranna et al., 2008; Shea and Lee, 2011; Holmgren et al., 2012). Direct evidence for this role of NF-H was provided by the inhibition of establishment of this slower-transporting NF population, the so-called stationary cytoskeleton, by deletion of the C-terminal domains of NF-H and NF-M (Rao et al., 2012). Additional experimental evidence for this role has been provided by studies in cultured neurons or neuronal cells following expression of mutagenized NF subunits and/or their relevant kinases, or intracellular delivery of specific antibodies, in which key manipulations could not only decrease, but also increase, NF-H-mediated interactions (Shea and Beermann, 1994; Yabe et al., 2001; Ackerley et al., 2003; Shea and Lee, 2011; Yuan et al., 2012; Lee et al., 2014). Herein, shRNA-mediated depletion of NF-H provides additional experimental evidence that NF-H provides stability to developing axonal neurites, and that this stability facilitates relatively early stages of axonal outgrowth. Notably, these short-term experiments conducted in culture avoided potential developmental compensation by NF-M for lack of NF-H (Sánchez et al., 2000; Shea and Chan, 2008). A number of factors substantially reduced the likelihood that shRNA treatment artifactually compromised axonal outgrowth and stabilization by inducing overall cytotoxicity, including (1) the lack of effect on levels of other NF subunits, tubulin or actin, (2) rescue of axonal neurite length following expression of exogenous NF-H while endogenous NF-H was inhibited, and (3) prevention of this rescue by simultaneous inhibition of exogenous along with endogenous NF-H.

shRNA directed against NF-H reduced the overall percentage of cells with axonal neurites during outgrowth and following colchicine treatment, indicating a requirement for NF-H in outgrowth and attainment of the degree of stabilization that provides resistance to retraction following colchicine treatment. Notably, shRNA treatment also reduced the mean length of neurites both during outgrowth and those surviving colchicine treatment. Notably, however, neurite length varies considerably among cells during differentiation (Shea and Beermann, 1994). Quantification of the distribution of neurite lengths prior to and following shRNA treatment demonstrated that shRNA treatment did not simply eliminate shorter neurites, but instead reduced the length of the entire spectrum of neurites (Table 2). Those neurites which were reduced to <1 somal diameter were responsible for the observed result decrease of cells scored as possessing neurites, but we observed elimination/reduction in frequency of even the longest neurites (e.g. >5 somal diameters).

**Fig. 6.**
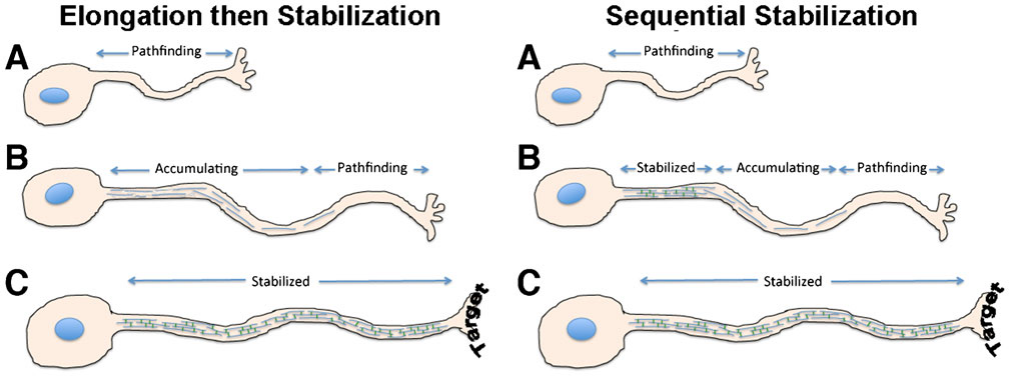
Model for axonal pathfinding and stabilization. In the conventional “elongation then stabilization” model, the axon stabilizes only after reaching its target. Based on our findings herein, we propose a “sequential stabilization” model, where more proximal regions of the growing axon progressively stabilize, and therefore physically support, continued distal pathfinding.

One interpretation of these findings is that axonal neurites of NB2a/d1 cells do not necessarily develop stable cytoskeletal networks along their entire length following a critical extent of elongation (e.g. >3 somal diameters), but rather that axonal neurites instead contain relatively stable regions and relatively labile regions. Extrapolation of these findings in cultured neuroblastoma cells to axonal elaboration *in situ* is consistent with the notion that developing axons likely undergo sequential stabilization, and that proximal stabilization supports continued pathfinding by labile, distal regions. These distal regions undergo stabilization, which supports further pathfinding. The entire axon would therefore stabilize only following synaptogenesis (Fig. 6). Axonal pathfinding and subsequent stabilization are generally considered to be separate phenomena. However, given that axons *in situ* can be thousands of times longer than the neuronal soma, many axons must traverse considerable distances to reach their target; it is indeed improbable that stabilization of the axon can be delayed until synaptogenesis is complete.

Our findings support a role for NF-H, and in particular interactions among cytoskeletal elements mediated by the NF-H C-terminal sidearm, in providing axonal stabilization, and that this stabilization supports overall axonal neurite elaboration. Radiolabel analyses demonstrated that shRNA treatment virtually halted NF-H synthesis and depleted steady-state levels in undifferentiated cells. By contrast, steady-state NF-H levels in differentiated cells were reduced by only 40%. Retention of this level of NF-H is likely to be due to a combination of C-terminal phosphorylation and its incorporation into the developing axonal cytoskeletal matrix, both of which result in slower turnover of NF-H (Shea and Lee, 2013; Lee et al., 2014). In this “sequential stabilization” model, the cytoskeletal dynamics that underlie elongation and stabilization therefore occur within the same developing axon, presenting the challenge of maintaining a temporal and spatial segregation of their respective dynamics. During development, a given axon may therefore have a crosslinked NF population in it most proximal region, followed by a region in which the NFs are accumulating, and finally a distal, NF-poor region where active elongation and pathfinding continues (Fig. 6). This model is consistent with our prior observations in developing axonal neurites in culture; while NFs containing newly-expressed (GFP-tagged) subunits were observed along the length of axonal neurites, their incorporation into the centrally-situated NF bundle, which consists of extensively cross-linked phospho-NFs and corresponds to the stationary cytoskeleton observed *in situ* (Yabe et al., 2001; Yuan et al., 2009), was more protracted and occurred in a proximal–distal manner (Yabe et al., 2001).

The phosphorylation events that mediate NF-H C-terminal phosphorylation and the interaction of NFs with each other and with other cytoskeletal elements is regulated by a complex hierarchy of kinase activities, including glycogen synthase kinase 3beta (GSK), p42–44 mitogen-activated protein kinase (MAPk), cyclin-dependent kinase 5 (cdk5) (Lee et al., 2014). Notably, growth cones (GCs) of pathfinding axons utilize these kinases to integrate signaling from multiple guidance cues. For example, nerve growth factor, which regulates GC activity, stimulates GSK via activation of p42–44 MAPk (Zhou et al., 2004; Goold and Gordon-Weeks, 2005). Similarly, brain derived neurotropic factor, which promotes GC filopodial dynamics and axonal branching, activates MAPk, while ephrin-A, which provokes GC collapse, inhibits MAPk (Yue et al., 2008; Meier et al., 2011). Conversely, cyclin-dependent kinase 5 (cdk5) is activated by tyrosine kinase activation in response to the GC collapse signal ephrin-A (Cheng et al., 2003). Similarly, p42–44MAP kinase promotes NF axonal transport, while cdk5 and GSK3 inhibit transport and promote axonal NF bundling (Chan et al., 2004; Shea et al., 2004; Kushkuley et al., 2009; Lee et al., 2014). Utilization of overlapping kinase cascades for GC-mediated pathfinding and NF-mediated stabilization not only eliminates any requirement for the neuron to restrict the distribution of key kinases or to invoke an entirely different kinase cascades, but also provides the possibility that a neuron could maintain temporal and spatial segregation of pathfinding and stabilization simply by the developmental delay in expression and transport of one key element (NF-H).

The present study focused on NFs, and in particular, NF-H. However, axonal outgrowth and maturation is also critically dependent upon MTs and their associated proteins. Axonal neurites develop a population of long, stationary MTs (Zhou and Cohan, 2004; Baas et al., 2006; Myers and Baas, 2007). NFs undergo MT-dependent axonal transport is dependent upon MTs, and resident axonal NFs align along these MTs within developing axons (Shea and Lee, 2013). In addition, the development of stability in these cells as evidenced by resistance to colchicine-mediated retraction is not due to NF-H in isolation, but rather is derived from undisclosed interaction(s) of phospho-NF-H with the MT-associated proteins MAP1B and tau (Shea and Beermann, 1994). Of interest would be to manipulate expression of these proteins, and that of NF-M along with NF-H, and monitor the impact on axonal neurite development.

## MATERIALS AND METHODS

NB2a/d1 cells were utilized in these studies due to their ease of transfection, including multiple transfections as required herein (Lee et al., 2014). It is recognized that these cells are a model rather than bona-fide neurons. However, cultured embryonic neurons are difficult to transfect. Cells were differentiated by treatment with dbcAMP, which induces elaboration of neurites bearing multiple characteristics of axons, including a robust population of axonal NFs (Yabe et al., 2001). Cells were transfected with a cocktail of 4 shRHAs corresponding to different coding regions of murine NF-H (Table 1). In additional experiments, cells were transfected shRNA corresponding to a C-terminally located untranslated region of murine NF-H (UTR-1), or CDS-4 alone, which corresponds to a portion of the coding sequence common to both murine and rat NF-H (Table 1).

### Transfection

For examination of the impact of shRNA treatment on axonal neurite outgrowth, cells were transfected with one or more shRNA clones (Table 1) using Lipofectamine as described (Chan et al., 2004) 48 hr prior to treatment with dbcAMP.

In some experiments, cells were cotransfected with a plasmid expressing rat NF-H conjugated to green fluorescent protein (GFP-H) (Yabe et al., 2001; Lee et al., 2014) along with one or more of the above shRNA plasmids, then differentiated with dbcAMP as above. Additional cells were transfected with the GFP-conjugated rat NF-H construct in which a stop codon (TGA) was introduced immediately after T492 (based on the numbering of Chin and Liem, 1990), which immediately precedes the first confirmed C-terminal phosphorylation site (S493; a GSK3β consensus site (S493) (Sasaki et al., 2002), using the following primers; 5′-GAGAAGAAGCAGCAACTACGTGACCCCCTGCAGA-3′ and 5′-TCTGCAGGGGGTCACGTAGTTGCTGCTTCTTCTC-3′. This early termination mutation was confirmed by dideoxy early termination sequencing service (Beckman Coulter Genomics, Danvers, MA).

### Quantification of neurite outgrowth and stabilization

Axonal neurite length was quantified as a function of respective somal diameter (Shea and Beermann, 1994; Dubey et al., 2004). For quantification of the percentage of cells possessing axonal neurites, cells were scored as positive if they possessed an axonal neurite >1 respective somal diameter; cells elaborating only filopodia ≤1 somal diameter in length were scored as lacking axonal neurites.

Following 7 days of continuous dbcAMP treatment, axonal neurites of NB2a/d1 cells undergo stabilization to the extent that they are resistant to retraction following colchicine treatment (Shea and Beermann, 1994). For examination of the impact of shRNA treatment on axonal stabilization, cells therefore received dbcAMP for 7 days followed by treatment with 1 µM colchicine for 2 hr (Shea and Beermann, 1994). In these latter experiments, cells were transfected with shRNA either 24 hr prior to this 7-day dbcAMP treatment or for the final 24 hr. Medium was replaced every 3 days; fresh dbcAMP and shRNA were included medium replacement.

### Electrophoresis and immunoblot analysis

Cells were homogenized in 50 mM Tris-HCl (pH 6.8) containing 1% Triton X-100, 5 mM EDTA, 1 mM PMSF and 50 µg/ml leupeptin and centrifuged (15,000 × g; 15 min). The resulting pellet was defined as the Triton-insoluble cytoskeleton, and the resulting supernatant was defined as the Triton-soluble fraction. Samples were normalized according to total protein, subjected to SDS-gel electrophoresis and transferred to nitrocellulose. Membranes were blocked with 5% BSA and 5 mM sodium fluoride in Tris-buffered saline containing 0.1% Tween-20 for 1 hr then incubated overnight at 4°C with antibodies directed against GFP (1:1000, Invitrogen), an antibody directed against all NFs regardless of phosphorylation state (R39), antibodies directed against NF-H (H3) and NF-M (M2) regardless of phosphorylation state (Jung and Shea, 1999), antibodies directed against NF phospho-epitopes [RT97 (generous gift of Dr. B. Anderton) and SMI-31 (Covance; Dedham, MA)], and antibodies directed against alpha-tubulin (DM1a, Sigma) and beta-actin (ABM: Richmond, BC, Canada). Membranes were washed with the same buffer then incubated with alkaline phosphatase-conjugated secondary antibodies for 1 hr at room temperature and developed using a NBT/BCIP substrate kit (Promega, Madison, WI). Immunoreactive species were quantified in digitized images of replicas using Image J; the background signal from an adjacent, identically-sized area in the identical lane was subtracted from each reactive species (Yabe et al., 2001). All samples to be compared were electrophoresed on the same gel, transferred to nitrocellulose and visualized simultaneously.

### Immunofluorescence

Cells grown on poly-L-Lysine-treated coverslips were fixed with 4% paraformaldehyde in phosphate-buffered saline (PBS; pH 7.4) for 10 minutes at room temperature, rinsed 2× in PBS (5 min/rinse), and blocked for 30 min in PBS containing 1% bovine serum albumin (BSA) and 2% normal goat serum. Cultures were then incubated overnight at 4°C in PBS containing 1% BSA and 1:100 dilutions of the above antibodies. Cultures were rinsed 3× with PBS, incubated for 30 min at 37°C in PBS containing 1% BSA and a 1:300 dilutions of appropriate secondary antibodies.
